# *Mucuna pruriens* in untreated Parkinson's disease in sub-Saharan Africa: A 12-month, multicenter, randomized, controlled trial

**DOI:** 10.1177/1877718X251383721

**Published:** 2025-11-21

**Authors:** Roberto Cilia, Momodou Cham, Vida Obese, Albert Akpalu, Emanuele Cereda, Elikem Ame-Bruce, Ruth Laryea, Serena Caronni, Fred Stephen Sarfo, Francesca Del Sorbo, Stanley Fahn, Gianni Pezzoli

**Affiliations:** 1 Parkinson and Movement Disorders Unit, Department of Clinical Neurosciences, 9328 Fondazione IRCCS Istituto Neurologico Carlo Besta, Milan, Italy; 2 743742 Richard Novati Catholic Hospital, Sogakope, Ghana; 3 259295 Komfo Anokye Teaching Hospital, Kumasi, Ghana; 4 285284 Korle Bu Teaching Hospital, Accra, Ghana; 5 Department of Medicine, 63533 University of Ghana Medical School, Accra, Ghana; 6 Clinical Nutrition and Dietetics Unit, 18631 Fondazione IRCCS Policlinico San Matteo, Pavia, Italy; 7 743742 Fondazione Pezzoli per la Malattia di Parkinson, Milan, Italy; 8 Parkinson Institute, ASST 18605 Gaetano Pini-CTO, Milan, Italy; 9 12294 Columbia University College of Physicians and Surgeons, New York, NY, USA

**Keywords:** *Mucuna pruriens*, Parkinson's disease, levodopa, accessibility, equality

## Abstract

**Background:**

Parkinson's disease (PD) causes disability and premature mortality if untreated. Limited access to levodopa in low- and middle-income countries leaves many patients undertreated. *Mucuna pruriens* (MP) is a leguminous plant that contains high concentrations of levodopa.

**Objective:**

To demonstrate the non-inferiority of long-term intake of MP powder in terms of safety and efficacy compared to standard levodopa *plus* dopa-decarboxylase inhibitor (LD + DDCI).

**Methods:**

In this 12-month, multicenter, randomized, open-label phase 2 trial, thirty-two untreated PD patients received levodopa monotherapy with MP powder -derived from roasted seeds without pharmacological processing- or standard LD + DDCI. Dosing was adjusted for body weight and disease stage, with MP doses further calibrated to account for the absence of a DDCI. We measured quality of life using the 39-item PD Questionnaire, motor and non-motor disability using the Movement Disorders Society updated version of the Unified PD Rating Scale (MDS-UPDRS) (parts I to IV) and the Non-Motor Symptoms Questionnaire. Safety measures included recording any adverse event and laboratory test.

**Results:**

MP powder improved quality of life, motor and non-motor symptoms over 12 months, demonstrating similar outcome to LD + DDCI on all endpoints. Adverse events were more frequent with MP (56% vs. 37.5%, p = 0.48), though the difference was not statistically significant. Most were mild, with only 12.5% leading to discontinuation.

**Conclusions:**

MP could be a cost-effective alternative for PD individuals with limited access to commercial levodopa formulations. To confirm long-term safety and efficacy, larger international multicenter, double-blind trials with extended follow-up (e.g. 24–36 months) and ethnically diverse cohorts are needed.

Registered at PACTR201611001882367

## Introduction

Parkinson's disease (PD) is the fastest-growing neurological disorder worldwide, currently affecting over six million people, with projections indicating that this number will steeply increase in the next two decades.^1,2^ This rise is particularly concerning in low- and middle-income countries (LMICs), where increasing life expectancy and demographic shifts are expected to lead to a significant rise in PD prevalence, reaching 300% in sub-Saharan African (SSA) by 2050.^2^ Despite this rising burden, access to effective PD treatment remains severely limited in many LMICs worldwide, where health services are poorly prepared to cope with the ageing of the population and the potential rise in environmental exposures.^1–3^

Levodopa combined with a dopa decarboxylase inhibitor (LD + DDCI), the cornerstone of PD therapy, is often unavailable or unaffordable in LMICs, leaving many patients undertreated.^4–8^ In a nation-wide survey in Ghana with data obtained from 121 pharmacies throughout 10 regions, LD + DDCI was available only in 11% of the pharmacies surveyed (ranging from 5% in public to 14.6% in private ones).^9,10^ The lack of adequate pharmacological management exacerbates disability, diminishes quality of life, and places an increasing strain on caregivers and healthcare systems. Addressing this gap on the essential pharmacological treatment is crucial for improving outcomes for PD in resource-limited settings.^10,11^

*Mucuna pruriens* (MP), a tropical leguminous plant growing abundantly in many LMICs and naturally rich in levodopa (with concentrations usually ranging between 4% and 6%), offers a promising alternative to conventional LD + DDCI.^12,13^ Several clinical studies, including open-label and randomized controlled trials (RCTs), have investigated the efficacy and safety of MP in PD.^13^ During the last 10 years, our team have assessed the use of MP powder obtained from roasted seeds using a low-cost preparation method without any pharmacological processing.^13–15^ Given its high levodopa content and accessibility in LMICs, it is crucial to assess whether MP can serve as a sustainable alternative to conventional levodopa-based therapy where access to standard pharmacological therapy remains a challenge.^12^

In the present multicenter study, we aimed to compare the efficacy and safety of MP with commercial LD + DDCI over 12 months and to generate clinical evidence on MP role as a potential first-line therapy in settings where access to conventional pharmacological treatment is limited. If successful, MP could reduce disparities in access to levodopa in resource-limited regions, improving motor and non-motor disability for millions of individuals with PD.

## Methods

*Study design*. This was a 12-month, multicenter, noninferiority, open-label, randomized, controlled phase 2 trial, comparing safety and efficacy of MP powder compared to levodopa plus DDCI in newly diagnosed untreated individuals with PD. The overall trial duration was 36 months and the treatment duration for each patient was 12 months. The trial started on February 2018 and was completed on June 2021.

### Participants and eligibility criteria

*Inclusion criteria*: (i) age range 30 to 80 years; (ii) diagnosis of clinically established PD according to MDS criteria^16^; (iii) newly diagnosed PD; (iv) never treated with levodopa (drug-naïve) or treated for ≤6 months during the disease course but levodopa discontinued since at least 3 months. *Exclusion criteria*: (i) dementia according to DSM-V criteria precluding the subject to provide written informed consent; (ii) clinically significant psychiatric illness (e.g. severe depression or psychosis); (iii) Hoehn and Yahr stage 5/5; (iv) severe, unstable medical conditions (e.g. neoplasms; unstable diabetes mellitus; heart, renal or liver failure) precluding the participation to the trial; (v) pregnancy.

*Enrollment*. Written informed consent was obtained from every patient; additional consent was requested for video recording and publication. Informed consent was translated into local Ghanaian dialect whenever required. The pseudonymization code assigned to each participant was generated upon signing the informed consent and followed a sequential numbering system specific to each of the three Ghanaian recruiting centers (GH-01-01 for Sogakope; GH-02-01 for Accra; GH-03-01 for Kumasi).

Prior to study initiation, the local raters were trained by neurologists with longstanding experience in movement disorders (R.C., D.S.F., S.F., G.P.) on how to perform neurological examination of individuals with PD and how to administer all scales and questionnaires. Local raters obtained the international certification on Good Clinical Practice as well as certification on Movement Disorders Society updated version of the Unified PD Rating Scale (MDS-UPDRS) rating by the International Parkinson and Movement Disorders Society.

*Timeline and activities.* Screening (week -6 to day -1, V0); Baseline visit (V1); 3-month visit (week 12 ± 7 days, V2); 6-month visit (week 26 ± 7 days, V3); 9-month visit (week 38 ± 7 days, V4); 12-month visit (week 52 ± 7 days, V5). During the *screening* phase, patients underwent a challenge with MP powder,^14^ and their compliance on the use of MP powder over a 12-month period was investigated with patients and caregivers. Specifically, we conducted interviews to assess the willingness and ability to adhere to the treatment regimen and to regular follow-up visits to the clinic, including potential barriers such as travel distance to the clinic. At the *baseline* visit, patients underwent a neurological examination, previously performed laboratory tests and electrocardiography were reviewed, scales and questionnaires were completed. Then, participants entered a dose-adjustment period lasting up to 3 weeks, during which treatment doses and number of daily intakes were optimized. The dose regimen of either MP or LD + DDCI was calculated according to body weight and disease stage including a titration phase.^13–15^ At each timepoint, patients were assessed and questionnaires completed. No dose adjustments were permitted during the maintenance phase and no ‘rescue’ therapy was allowed.^15^ Comprehensive *laboratory tests* were performed in all individuals, according to our previous clinical trial.^15^

*Randomization*. Eligible patients were randomized in a 1:1 allocation ratio into the two treatment arms (MP versus LD + DDCI) using a centralized randomization system. The randomization list was computer-generated using dedicated software (https://ctrandomization.cancer.gov/).

*Interventions.* The investigational treatment was MP powder made directly from roasted seeds, without any pharmaceutical processing or additional compounds^17^ and administered dissolved in half a glass of water.^13–15^ In case of any gastrointestinal complaint, advice was given to use a modified MP preparation using supernatant water, a method that involves consuming the liquid phase after sedimentation of the MP powder.^13,15^ We used one local MP ecotype at the three study sites. To optimize the dose equivalence between the two arms, we measured *a priori* the concentration of levodopa,^13,17^ which was 6.3% in dried seeds. Videos on the low-cost preparation method of MP powder are freely available online,^13,17^ whereas details on MP preparation, storage and delivery to patients are provided elsewhere.^13^ According to prior studies in early 70s by Marsden and colleagues, who compared levodopa alone versus levodopa + carbidopa^18^ and to our previous study including pharmacokinetics of MP versus levodopa alone and LD + DDCI,^14^ the average bioavailability of levodopa alone is approximately 5-fold higher than levodopa combined with DDCI. LD + DDCI was provided as levodopa + benserazide 200 + 50 mg tablets and supplied free of charge.^19^

After completing the trial, all patients were provided with standard levodopa treatment free of charge, which all surviving participants still use.^19^ The use of MP is permitted only under medical supervision within clinical trials.^13^

### Objectives and outcome measures

*Objectives.* The purpose of this study was (i) to assess the efficacy of MP in improving motor symptoms compared to LD + DDCI; (ii) to evaluate the safety and tolerability of MP over a 12-month period, particularly regarding Adverse events (AEs) and discontinuation rates. By addressing these key areas, this study aimed to provide crucial insights into the potential of MP as an accessible and effective PD therapy for newly diagnosed PD in SSA.

#### Outcome measures

- The primary efficacy outcome measure was the difference in mean change at 12 months in quality of life between the two treatments, as assessed using the summary index of the 39-item PD quality of life questionnaire (PDQ-39).^20^
- Secondary outcome measures included: (i) PDQ-39 subitems; (ii) non-motor and motor experiences of daily living, motor examination and motor complications were assessed by the MDS-UPDRS parts I, II, III and IV and the Hoehn & Yahr stage (H&Y)^21,22^; (iii) severity of non-motor symptoms assessed by the validated non-motor symptoms questionnaire (NMSQ).^23^

*Safety.* Participants were monitored for AEs on day 7, day 14, day 21 by telephone and on day 28 for the first in-person follow-up visit. Then all patients were visited to check for AEs every month, whereas they were assessed on months 3, 6, 9 and 12. They were also informed to call in and report any unusual symptoms to the trial team via phone in-between visits if necessary. All symptoms which meet the criteria for AE, including serious adverse events (SAE), were recorded and graded. AEs were followed up until resolution, with the appropriate treatment and care being given to the affected participant at no extra cost to them. The Investigational Medicinal Product (IMP) could have been discontinued prematurely or temporarily halted if any unacceptable findings were identified, such as: (i) the occurrence of a SAE that was considered at least possibly related to the IMP; (ii) termination of the study; (iii) upon the subject's request (withdrawal of consent).

### Standard protocol approvals, registrations, patient consents, trial registration

This study was performed in accordance with good clinical practice and the Declaration of Helsinki. Ethical approval was obtained by the three recruiting centers and informed consent was obtained by all participants in the language of their choosing. All data collected were pseudonymized with the use of participant ID numbers. This trial was registered on Cochrane Central Register of Controlled Trials (PACTR, identifier 201611001882367).

### Statistical analysis

The study was initially sized to compare the efficacy of MP and LD + DDCI, as well as to enable to refine study protocols and maximize the chances of identifying problems that may arise in a large multi-center trial. We originally planned to enroll at least 90 patients [45 per treatment arm - considering drop-out rate of 20%^24^ to detect clinically meaningful difference in the summary index of the PDQ-39 [effect size of 0.5^25^; power of 80%; two-tailed type-I error of 20%]. Therefore, given the difficulties arose in recruitment due to the SARS-CoV-2 pandemic, we resized the trial to address the non-inferiority of MP as compared to LD + DDCI. Considering a clinically meaningful difference in PDQ-39 of 4 points,^26^ we assumed that the non-inferiority margin is chosen to be 2 points, supposing a true mean difference of 0 points and a standard deviation of 18 points. For achieving an 80% power at the 5% level of significance, with equal allocation (1:1) and a drop-out rate of 20%, we calculated a sample size of 32 patients (16 per treatment arm).

Safety and efficacy analyses were conducted on all patients randomized and those completing the study, respectively. Descriptive statistics were provided for continuous (mean and standard deviation or median and interquartile range [IQR, 25th–75th percentile]) and categorical (count and percentage) variables. The primary outcome measure and all continuous endpoints were compared between interventions using a general linear regression model for repeated measures. Crude and adjusted (for baseline values) mean differences between treatments were computed accordingly. Analyses over multiple time points were also performed. Finally, the frequencies in adverse events were compared using the Fisher's exact test.

Data were analyzed blinded to treatment using the software program SPSS (Windows Release 17.0; SPSS Inc, Chicago, IL, USA). A two-sided p-value of <0.05 was set as significant.

## Results

**Figure 1.**
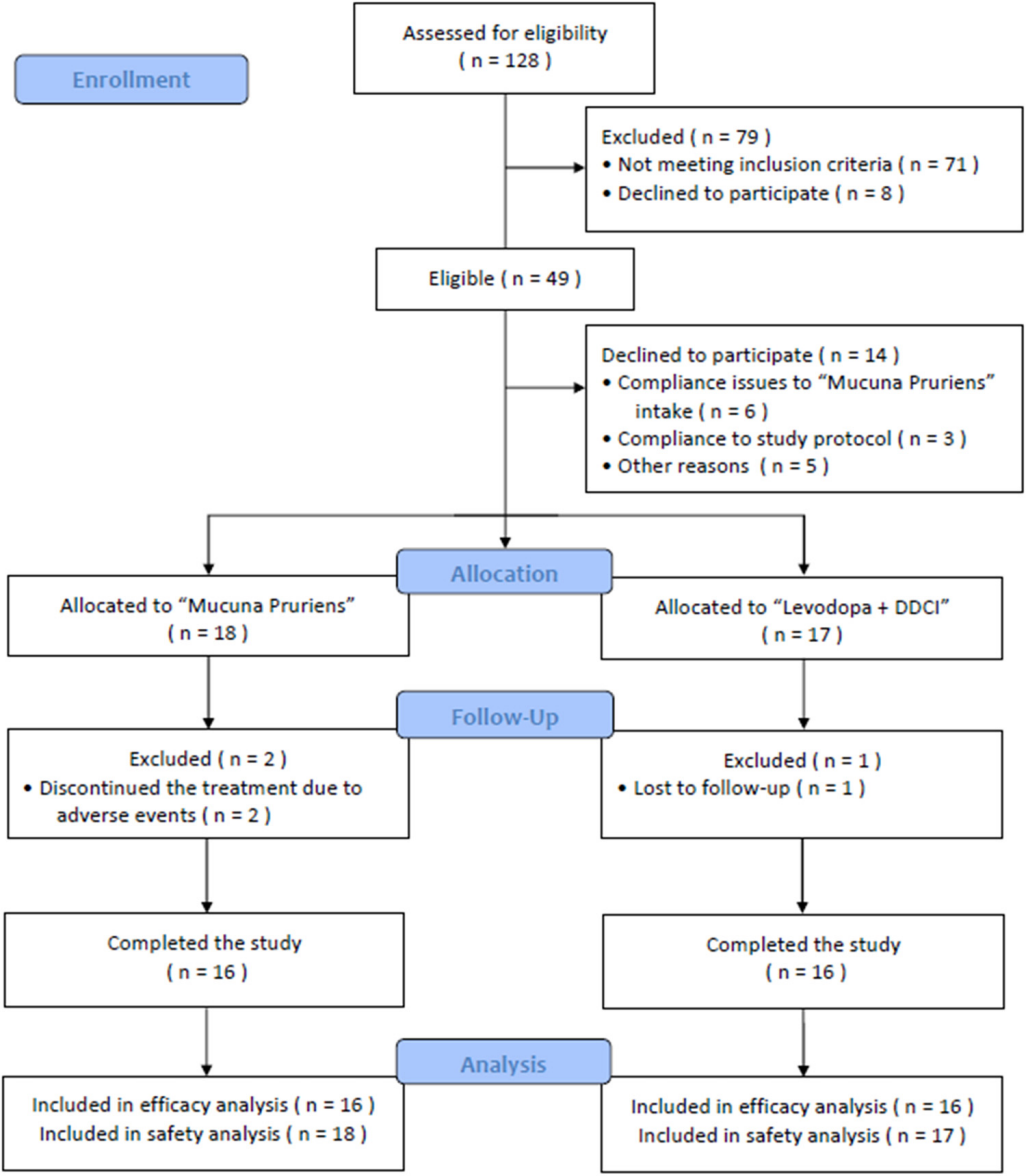
Study flow chart.

*Study population.* A total of 128 individuals were screened at the three recruiting centers, of whom 79 were excluded, mainly because they were already on stable levodopa therapy. Fourteen participants withdrew consent prior to randomization: six due to difficulties adhering to MP powder intake (e.g. preferring tablets over powder), three due to challenges complying with the study protocol (e.g. difficulty attending regular hospital visits every three months), and five for other reasons. The study flowchart (consort diagram) is summarized in Figure 1.

Thirty-five PD patients (20 males, mean PD duration 3.8 ± 3.4 years) were randomized. Demographic and clinical features at baseline were similar between the two groups (Table 1). The study population exhibited heterogeneity in both disease duration and clinical severity, primarily driven by delayed diagnoses, with some individuals being diagnosed up to 10–12 years after the initial onset of symptoms. During the maintenance phase, daily dosage of MP powder was 36.7 ± 5.0 grams (0.62 ± 0.13 g/kg), subdivided in 3.15 ± 0.38 intakes/day. Considering the 6.3% levodopa concentration in the MP ecotype used, the estimated mean daily amount of levodopa was 2.3 grams, estimated to correspond to 462 ± 63 mg/day of LD + DDCI in terms of CNS availability. Mean LD + DDCI dose was 410 ± 117 mg/day, subdivided in 3.30 ± 0.5 intakes/day. There was no difference in protocol adherence or treatment discontinuation rates across the study centers.

**Table 1.** Baseline demographic and general clinical features by treatment allocation (efficacy population).

Features	*Mucuna pruriens*	Levodopa + DDCI
	(N = 16)	(N = 16)
Men, n (%)	11 (68.7)	9 (56.2)
Age (years), Mean (SD)	63.9 (9.2)	59.7 (9.0)
Disease duration (years), Mean (SD)	3.6 (3.4)	3.9 (3.6)
Tremor-dominant phenotype, n (%)	15 (94)	14 (87.5)
PDQ-39 Summary Index, Mean (SD)	38.9 (19.1)	37.6 (16.9)
PDQ-39 Mobility Index, Mean (SD)	51.9 (23.6)	46.7 (23.8)
MDS-UPDRS part I, Mean (SD)	11.9 (8.0)	10.6 (7.8)
MDS-UPDRS part II, Mean (SD)	18.0 (8.6)	19.7 (9.1)
MDS-UPDRS part III, Mean (SD)	50.8 (21.3)	58.1 (14.2)
MDS-UPDRS part IV, Mean (SD)	0.2 (0.5)	0.5 (1.2)
MDS-UPDRS Total score, Mean (SD)	80.9 (33.5)	88.9 (26.6)
Hoehn and Yahr stage, Mean (SD)	2.5 (0.7)	2.8 (0.6)
NMSQ Total score, Mean (SD)	11.1 (7.2)	8.6 (5.8)

Abbreviations: MDS-UPDRS: Movement Disorders Society Unified Parkinson's Disease Rating Scale; NMSQ: Non-Motor Symptoms Questionnaire; PDQ-39: PD Questionnaire on Quality of Life (39 items).

### Efficacy measures

**Figure 2.**
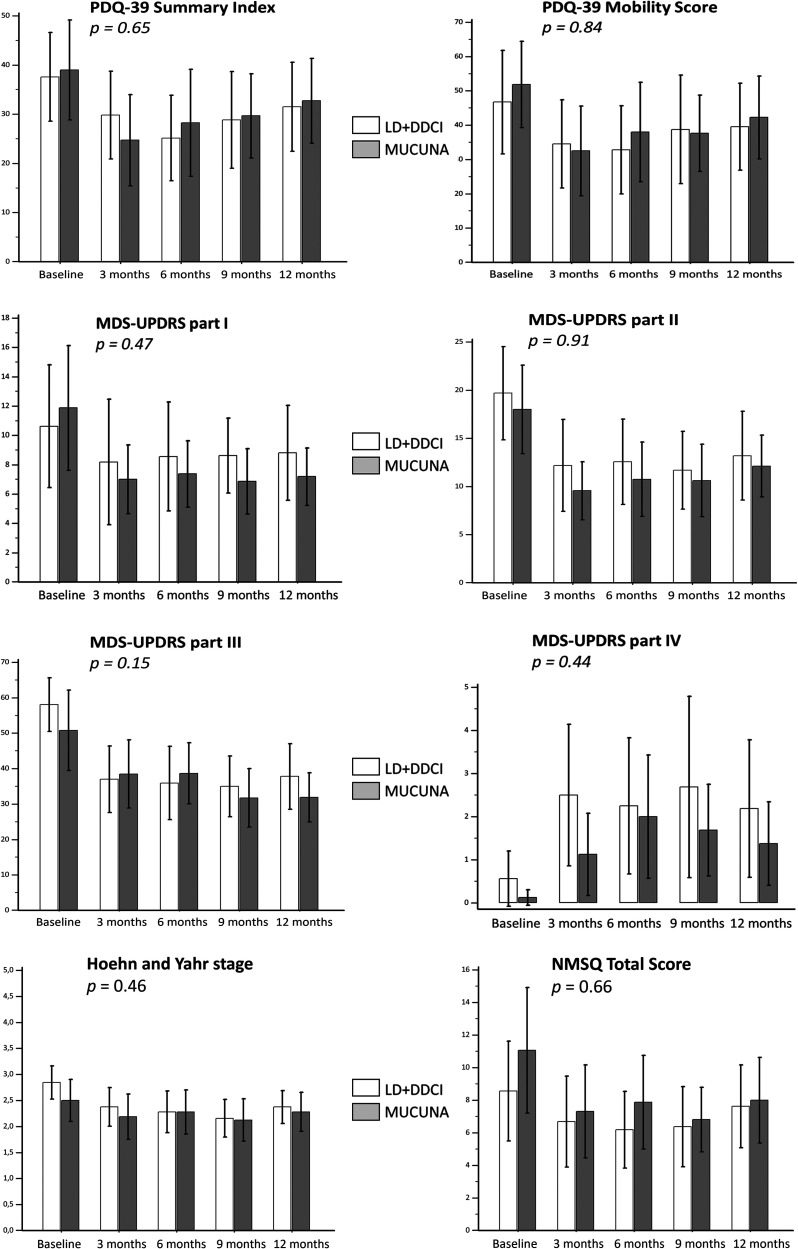
Mean scores (±standard deviation) of efficacy primary and secondary endpoints at all time points by study intervention.

Both treatments led to significant improvements in motor and non-motor symptoms, as well as quality of life, after 12 months of treatment. MP was non-inferior to LD + DDCI across all primary and secondary efficacy endpoints, with no statistically significant differences observed between groups at all time points (Table 2, Figure 2).

**Table 2.** Change (mean difference and 95%CI) in primary and secondary outcome variables in the efficacy population.

Outcomes	*Mucuna pruriens* Within-group change	Levodopa + DDCI Within-group change	Between-group difference (crude)	P-value	Between-group difference (adjusted)^a^^,^^b^	P-value
	(N = 16)	(N = 16)				
Primary						
PDQ-39 Summary Index	−6.3 (−15.9 to 3.4)	−6.1 (−13.8 to 1.7)	−0.2 (−12.0 to 11.7)	0.98	0.5 (−9.6 to 10.7)	0.90
PDQ-39 Mobility Index	−9.6 (−23.8 to 4.5)	−7.2 (−21.1 to 6.7)	−2.4 (−21.4 to 16.6)	0.80	−0.7 (−16.2 to 14.7)	0.92
Secondary						
MDS-UPDRS part I	−4.6 (−8.4 to −0.9)^c^	−1.8 (−5.2 to 1.6)	−2.9 (−7.7 to 2.0)	0.24	−2.0 (−5.2 to 1.1)	0.19
MDS-UPDRS part II	−5.9 (−9.5 to −2.3)^c^	−6.5 (−10.8 to −2.1)^c^	0.6 (−4.8 to 6.0)	0.82	−0.2 (−4.7 to 4.2)	0.91
MDS-UPDRS part III	−18.9 (−28.2 to −9.6)^c^	−20.3 (−27.8 to −12.7)^c^	1.4 (−10.1 to 12.8)	0.81	−2.4 (−11.9 to 7.1)	0.61
MDS-UPDRS part IV	1.2 (0.2 to 2.3)^c^	1.6 (0.0 to 3.2)^c^	−0.4 (−2.2 to 1.4)	0.68	−0.6 (−2.5 to 1.2)	0.48
MDS-UPDRS Total	−28.2 (−42.5 to −13.9)^c^	−26.9 (−39.4 to −14.4)^c^	−1.3 (−19.5 to 16.9)	0.89	−5.0 (−20.6 to 10.5)	0.51
NMSQ Total score	−3.1 (−6.8 to 0.7)	−0.9 (−3.1 to 1.3)	−2.1 (−6.3 to 2.0)	0.30	−0.6 (−3.7 to 2.5)	0.69

Abbreviations: MDS-UPDRS: Movement Disorders Society Unified Parkinson's Disease Rating Scale; NMSQ: Non-Motor Symptoms Questionnaire; PDQ-39: PD Questionnaire on Quality of Life (39 items).

^a^ A negative value represents an higher improvement in favor of the treatment with *Mucuna pruriens*.

^b^ Model adjusted for baseline values.

^c^ Within-group change significant at the 5% level.

The primary outcome, PDQ-39 summary index, improved similarly in both groups over the study period. The adjusted mean difference between MP and LD + DDCI at 12 months was 0.5 points (95% CI: −9.6 to 10.7; p = 0.90), supporting non-inferiority of MP. Notably, MP was associated with a slightly greater but non-significant improvement at the 3-month follow-up visit. Improvements were also observed in the PDQ-39 mobility domain in both arms, again with no significant between-group differences.

Regarding motor signs, both groups exhibited significant improvements in MDS-UPDRS part III scores: −18.9 (95% CI: −28.2 to −9.6) in the MP group and −20.3 (95% CI: −27.8 to −12.7) in the LD + DDCI group. The adjusted between-group difference was not statistically significant (–2.4; 95% CI: −11.9 to 7.1; p = 0.61). In three illustrative cases, previously untreated individuals with advanced PD with axial complications experienced marked improvement of motor signs following MP (Online Video). Concerning motor and non-motor experiences of daily living, similar findings were observed in MDS-UPDRS parts I and II, with within-group improvements reaching statistical significance in both groups. MDS-UPDRS part IV scores increased slightly in both groups (+1.2 in MP; +1.6 in LD + DDCI), reflecting the natural course of motor complications over time, yet the between-group difference remained nonsignificant (p = 0.48). Non-motor symptoms, as assessed by the NMSQ total score, improved in both MP and LD + DDCI arms (–3.1 vs. −0.9, respectively; adjusted p = 0.69). No significant differences were found in H&Y stage progression between the two groups over the follow-up period.

### Safety measures

AEs occurred in both treatment arms, with a similar frequency in the MP group (12/18, 66.7%) compared to the LD + DDCI group (13/17, 76.5%; p = 0.71; Table 3). Most AEs were mild to moderate in intensity and required treatment discontinuation in two individuals in the MP group (12.5%) due to intolerance—one because of persistent nausea and one due to diarrhea. In the MP group, the most reported AEs were gastrointestinal symptoms, particularly nausea (4 cases), followed by transient dizziness (2 cases), daytime somnolence, diarrhea and dyskinesias (1 case each). No SAEs were reported in either treatment arm.

**Table 3.** Adverse events recorded during the clinical trial.

Adverse Events	(A) Treatment-emergent AEs^a^			(B) Timing of AEs		(C) AEs leading to discontinuation	
	Levodopa + DDCI	*Mucuna Pruriens*	*p-value*	Levodopa + DDCI	*Mucuna Pruriens*	Levodopa + DDCI	*Mucuna Pruriens*
	(N = 17)	(N = 18)					
Any AE^b^	13	12	0.71	T1 (1)	T1 (5)	0	2
Any SAE	0	0	-	-	-	0	0
Nausea/Vomiting	2	4	0.65	T3 (2)	T1 (4), T2 (1), T3 (1), T5 (1)	0	0
Dizziness	1	2	0.99	T1 (1)	T1 (1), T2 (1)	0	0
Daytime Somnolence	2	1	0.99	T2 (1), T4 (1)	T4 (1)	0	1
Diarrhoea	0	1	0.99	-	T4 (1)	0	1
Orthostatic Hypotension	1	0	0.99	T2 (1)	-	0	0
Worsening of PD	0	0	-	-	-	0	0
Dyskinesias	0	1	0.99	-	T2 (1)	0	0
Psychiatric disturbances^c^	0	0	-	-	-	0	0
Alteration in laboratory tests	7	3	0.15	T1 (7)	T1 (3)	0	0

Abbreviations: AEs: Adverse events; PD: Parkinson's disease; SAE: Serious adverse event.

^a^ Number of patients reporting at least one adverse event; some patients reported more than one AE.

^b^ One patient may be represented at different time points if the AE persists.

^c^ Including hallucinations/psychosis, depression, addictive disorders and/or impulse control disorders.

Abnormalities in laboratory parameters were more frequently observed in the LD + DDCI group (7 patients) than in the MP group (3 patients), though the difference was not significant (p = 0.15). These changes remained within twofold of the reference range and were not clinically significant in any case. At 12 months, routine laboratory analyses (Supplementary Table 1) showed a mild increase in ferritin, vitamin B12, and folate levels in the MP group compared to LD + DDCI. Creatinine and eGFR values remained within normal range for all patients, though slightly higher creatinine levels and reduced eGFR were noted in the LD + DDCI group. No significant hepatotoxicity or hematological abnormalities were observed in either group.

## Discussion

In this randomized, controlled study, we provide the first evidence supporting the long-term use of MP powder as a feasible and effective alternative to conventional levodopa therapy in previously untreated individuals with PD living in Ghana. Over the 12-month treatment period, MP demonstrated similar safety profile and efficacy to LD + DDCI on multiple clinical endpoints, including motor and non-motor symptoms, as well as quality of life. Notably, no significant differences emerged between the two treatment groups on any of the outcome measures, and the study met its predefined non-inferiority criteria. Aligned with our primary aim to identify a sustainable treatment option for indigent PD patients, we focused our recruitment on newly diagnosed individuals or those who had initiated levodopa-based therapy but were unable to maintain it due to limited availability or affordability.

This study provides the first long-term efficacy data in drug-naïve PD patients, which reinforce and extend previous short-term studies that focused on patients with advanced disease in rural Bolivia^13–15^ and recently replicated in a small PD cohort in Thailand.^27^ MP powder led to significant within-group improvements in motor performance (MDS-UPDRS part III) and daily functioning (part II), as well as reductions in non-motor symptoms (NMSQ scores). While both treatment groups showed comparable improvements in quality of life (PDQ-39 Summary Index), MP showed a trend toward slightly greater benefit in certain domains (e.g. emotional well-being, stigma), although these differences did not reach statistical significance. While the relatively small sample size may limit the statistical power to definitively establish non-inferiority of MP compared to LD + DDCI, the illustrative cases presented in the online video offer compelling evidence supporting the therapeutic efficacy of MP in alleviating motor symptoms and enhancing quality of life, particularly in individuals at advanced PD stages where disability is most pronounced.

An equally important secondary endpoint was the long-term safety profile of MP powder. In this study, the overall tolerability of MP was generally acceptable, and its safety profile was comparable to that of LD + DDCI. Although gastrointestinal AEs occurred slightly more frequently in the MP arm, the difference was not significant and most were mild and transient. This favorable outcome is noteworthy considering that MP lacks a DDCI, a factor that may explain the trend toward increased gastrointestinal side effects -primarily nausea and vomiting- observed in our study, though these were not statistically significant. Our results aligned with previous pharmacokinetics studies that demonstrated that MP achieves plasma levodopa concentrations and ON-state durations comparable to LD alone, albeit requiring 5-fold higher doses. Only two patients (12.5%) discontinued MP due to intolerance, reflecting a low dropout rate over 12 months. This contrasts with a prior Bolivian study where 50% discontinued due to gastrointestinal side effects.^15^ In that study, all patients who withdrew were subsequently transitioned to a modified MP preparation using supernatant water -a method involving the consumption of the liquid phase after sedimentation of MP powder- resulting in improved tolerability and continued clinical benefit. To improve the tolerability of MP, we integrated this approach into our protocol for patients showing early signs of intolerance, which likely enhanced overall adherence compared to our previous trial.^15^ This simple, low-cost method, as outlined in recent guidelines, represents a practical strategy to reduce side effects and support long-term MP use in low-resource settings.^13^ Additionally, through a progressive ‘learning curve’, we developed a pragmatic strategy to fine-tune daily dosing that effectively balanced efficacy and tolerability, minimizing the risk for gastrointestinal discomfort while optimizing motor response^13,15^ (as shown in the paradigmatic cases featured in the online video). Importantly, no serious AEs were observed. Laboratory monitoring further confirmed that daily intake of MP powder holds premise to be safe in the long-term, as it was not associated with hepatic, renal, or hematologic toxicity, echoing safety findings from our previous 16-week trial.^15^

*Limitations and future perspectives.* There are limitations to acknowledge. First, the sample size was relatively small, as expected for a phase 2 trial. In part, this was due to the study design focusing on newly diagnosed cases with clear barriers to conventional pharmacotherapy, which led to a substantial reduction of the study population from 128 to just 35 cases who were randomized. However, the ethical implications of our decision are clear: we did not alter stable ongoing LD + DDCI treatments in well-managed patients, but rather limited study participation to a representative cohort of untreated individuals who represent the target population for whom MP could offer a meaningful alternative. Additionally, the COVID-19 pandemic limited access to the clinics, reducing recruitment opportunities. Second, although the open-label design may have introduced bias, the randomized controlled design increased the robustness of its findings. The consistency and magnitude of the clinical benefits we observed strongly justify further investigation in larger multicenter trials, such as the ongoing project “Transforming Parkinson's Care in Africa (TraPCAf)” aimed at expanding access to affordable PD treatments across sub-Saharan Africa.^28^ Third, this trial involved only Ghanaian patients and, albeit the effects largely overlapped with those we obtained in Bolivia^14,15^ and those reported in Thailand,^27^ generalizability to other ethnicities and regions needs to be verified. Finally, although no serious AEs or major laboratory abnormalities were observed, caution remains warranted regarding unsupervised MP use, given the potential for misuse and inconsistent dosing.^13^

The promising results of this trial provide a strong foundation for further clinical development of MP as a sustainable treatment option for PD in underserved regions. Future studies should prioritize larger, double-blind randomized controlled trials with open-label extension covering a longer period (e.g. 24–36 months) to confirm the long-term efficacy and safety of MP in diverse PD populations and across different MP ecotypes. Pharmacokinetic assessment and follow-up longer than 12 months are needed to fully describe the complication rate of using high-dose levodopa without DDCI for prolonged periods in relation to its exact cumulative exposure and bioavailability compared to equivalent doses of standard levodopa. This approach will also allow comparison between the effects of long-term use of MP with those of early levodopa studies in clinical settings.^18,29,30^ In our studies conducted in Bolivia^14,15^ and Ghana, we selected a single locally available MP ecotype to minimize potential safety concerns. Comparative pharmacokinetic studies evaluating different MP formulations -such as roasted powder versus supernatant water- are currently underway to optimize tolerability and dosing strategies. Lastly, regulatory frameworks should be considered to support the standardized use of MP, ensuring both clinical safety and equitable access for populations where pharmaceutical levodopa remains unaffordable or unavailable.

## Conclusions

This study preliminary provides evidence that long-term administration of MP powder as monotherapy over a 12-month period is non-inferior to commercial levodopa therapy in terms of efficacy, with an acceptable safety profile, in previously untreated patients with PD. These findings add to previous evidence on independent cohorts in non-African countries (such as Bolivia^15^ and Thailand^27^) and support the role of MP as a viable, locally available, and sustainable alternative to conventional levodopa formulations in low-resource settings globally. Cost-effectiveness and implementation strategies within public health systems should be evaluated accordingly. The economic impact could be substantial, as local cultivation could enhance economic self-sufficiency. Implementation strategies should prioritize standardized protocols for cultivation, processing and storage, alongside clinician training and patient education to ensure safe use.^13^ Initiatives like the TraPCAf project^28^ can support evidence-based policy-making and regulatory approval, paving the way for MP inclusion in national treatment guidelines.

To conclusively establish the long-term efficacy, safety and tolerability of MP and to ensure generalizability and external validation, further international multicenter studies should enroll larger, ethnically diverse cohorts and adopt a rigorous double-blind randomized controlled design with extended follow-up of 24 to 36 months.

## Supplemental Material

sj-docx-1-pkn-10.1177_1877718X251383721 - Supplemental material for *Mucuna pruriens* in untreated Parkinson's disease in sub-Saharan Africa: A 12-month, multicenter, randomized, controlled trial Supplemental material, sj-docx-1-pkn-10.1177_1877718X251383721 for *Mucuna pruriens* in untreated Parkinson's disease in sub-Saharan Africa: A 12-month, multicenter, randomized, controlled trial by Roberto Cilia, Momodou Cham, Vida Obese, Albert Akpalu, Emanuele Cereda, Elikem Ame-Bruce, Ruth Laryea, Serena Caronni, Fred Stephen Sarfo, Francesca Del Sorbo, Stanley Fahn and Gianni Pezzoli in Journal of Parkinson's Disease
