# Supplementary material for: Mucuna pruriens in untreated Parkinson's disease in sub-Saharan Africa: A 12-month, multicenter, randomized, controlled trial
Source: J Parkinsons Dis. 2025 Nov 21;16(1):99–109. doi: 10.1177/1877718X251383721 (PMC13347555; doi:10.1177/1877718X251383721)
Supplement: sj-docx-1-pkn-10.1177_1877718X251383721 - Supplemental material for Mucuna pruriens in untreated Parkinson's disease in sub-Saharan Africa: A 12-month, multicenter, randomized, controlled trial [file sj-docx-1-pkn-10.1177_1877718X251383721.docx]

**Supplementary Table 1.** Laboratory test results [mean (standard deviation)]

| Test (normal range)ᵃ | *Mucuna Pruriens* | Values outside the normal range (N) | Levodopa + DDCI | Values outside the normal range (N) |
| --- | --- | --- | --- | --- |
| **RBC** (3.5-5.0 10⁶/mL) | 4.7 (0.7) | 0 | 4.8 (0.6) | 0 |
| **Hb** (11-16 g/dL) | 14.2 (0.7) | 0 | 13.8 (1.4) | 0 |
| **MCV** (80-100 fL) | 88.9 (6.3) | 1 | 87.3 (5.5) | 1 |
| **WBC** (4-10 10³/mL) | 5.0 (0.7) | 0 | 6.2 (2.3) | 1 |
| **PLT** (140-400 10³/mL) | 230.6 (50.8) | 0 | 222.0 (55.0) | 1 |
| **Folates** (2.7-34 nmol/L) | 21.6 (11.5) | 1 | 16.3 (6.3) | 0 |
| **Vitamin B12** (pmol/L) | 271.1 (95.4) | 0 | 238.9 (143.5) | 0 |
| **Ferritin** (ng/mL) | 50.9 (29.9) | 0 | 76.6 (80.3) | 0 |
| **Urea** (mmol/L) | 3.1 (1.0) | 0 | 3.8 (1.4) | 0 |
| **Creatinine** (44-124 µmol/L) | 72.0 (18.6) | 0 | 87.5 (29.2) | 2 |
| **eGFR** (mL/min) | 86.4 (7.0) | 0 | 74.0 (16.2) | 2 |
| **ALT** (<40 U/L) | 15.0 (3.8) | 0 | 15.7 (8.6) | 0 |
| **AST** (<35 U/L) | 21.2 (3.9) | 0 | 23.0 (8.44) | 0 |
| **GGT** (7-51 U/L) | 30.4 (8.1) | 0 | 33.4 (13.8) | 1 |
| **Bilirubin Total** (µmol/L) | 12.0 (4.4) | 0 | 11.4 (2.9) | 0 |
| **Bilirubin Direct** (µmol/L) | 4.0 (1.1) | 0 | 4.1 (1.2) | 0 |
| **Alkaline Phosphatase** (U/L) | 90.0 (38.8) | 0 | 107.0 (22.0) | 0 |
| **Na⁺** (136-145) | 140.5 (2.3) | 0 | 140.2 (4.3) | 1 |
| **K⁺** (3.5-5.1) | 4.3 (0.6) | 0 | 4.3 (0.6) | 0 |
| **Cl⁻** (90-109) | 104.5 (2.4) | 0 | 101.8 (5.7) | 0 |
| **Total Proteins** (6.0-8.0 g/L) | 7.5 (0.7) | 1 | 7.7 (0.4) | 2 |
| **Albumin** (g/L) | 44.1 (4.2) | 0 | 45.0 (3.9) | 0 |
| **Alpha 1** (g/L) | 1.8 (0.3) | 0 | 1.7 (0.4) | 0 |
| **Alpha 2** (g/L) | 4.2 (0.7) | 0 | 6.9 (1.7) | 0 |
| **Beta 1** (g/L) | 5.0 (0.7) | 0 | 5.0 (0.7) | 0 |
| **Beta 2** (g/L) | 2.8 (0.6) | 0 | 2.9 (0.8) | 0 |
| **Gamma-Globulins** (g/L) | 13.9 (5.1) | 0 | 15.3 (2.9) | 0 |

*Abbreviations:* **ALT**, Alanine aminotransferase; **AST**, Aspartate aminotransferase; **eGFR**, estimated glomerular filtration rate; **GGT**, Gamma-glutamyl transferase; **Hb**, Hemoglobin; **MCV**, mean corpuscular volume; **PLT**, platelets; **RBC**, red blood cells; **WBC**, white blood cells.

*ᵃ Normal range values are in parentheses.*
